# Seasonal Changes in Gut Microbiota Diversity and Composition in the Greater Horseshoe Bat

**DOI:** 10.3389/fmicb.2019.02247

**Published:** 2019-10-01

**Authors:** Guohong Xiao, Sen Liu, Yanhong Xiao, Yue Zhu, Hanbo Zhao, Aoqiang Li, Zhongle Li, Jiang Feng

**Affiliations:** ^1^Jilin Provincial Key Laboratory of Animal Resource Conservation and Utilization, Northeast Normal University, Changchun, China; ^2^Institute of Resources and Environment, Henan Polytechnic University, Jiaozuo, China; ^3^School of Life Sciences, Jilin Agricultural University, Changchun, China

**Keywords:** hibernation, gut microbiota, fasting, diet, *Rhinolophus ferrumequinum*

## Abstract

A large number of microorganisms colonize the intestines of animals. The gut microbiota plays an important role in nutrient metabolism and affects a number of physiological mechanisms in the host. Studies have shown that seasonal changes occur in the intestinal microbes of mammals that hibernate seasonally. However, these studies only focused on ground squirrels and bears. It remains unclear how hibernation might affect the intestinal microbes of bats. In this study, we measured microbial diversity and composition in the gut of *Rhinolophus ferrumequinum* in different periods (early spring, early summer, late summer, torpor, and interbout arousal) using 16S ribosomal RNA gene amplicon sequencing and PICRUSt to predict functional profiles. We found seasonal changes in the diversity and composition of the gut microbes in *R. ferrumequinum*. The diversity of gut microbiota was highest in the late summer and lowest in the early summer. The relative abundance of *Proteobacteria* was highest in the early summer and significantly lower in other periods. The relative abundance of *Firmicutes* was lowest in the early summer and significantly increased in the late summer, followed by a significant decrease in the early winter and early spring. The relative abundance of *Tenericutes* was significantly higher in the early spring compared with other periods. The results of functional prediction by PICRUSt showed seasonal variations in the relative abundance of metabolism-related pathways, including lipid metabolism, carbohydrate metabolism, and energy metabolism. Functional categories for carbohydrate metabolism had significantly lower relative abundance in early winter-torpor compared with late summer, while those associated with lipid metabolism had significantly higher relative abundance in the early winter compared with late summer. Overall, our results show that seasonal physiological changes associated with hibernation alter the gut microbial community of *R. ferrumequinum*. Hibernation may also alter the metabolic function of intestinal microbes, possibly by converting the gut microflora from carbohydrate-related to lipid-related functional categories. This study deepens our understanding of the symbiosis between hibernating mammals and gut microbes.

## Introduction

The continuous co-evolution of mammals and their gut microbes forms a complex relationship that provides benefits for both partners (Ley et al., 2008a, b). The gut microbiome of animals is intimately linked to host physiology, nutrition, and metabolism (Hooper et al., 2012; Human Microbiome Project Consortium, 2012; Sommer and Backhed, 2013). The microbiome enhances resistance to pathogen colonization, affects the structure and function of the gut, drives the development of the immune system, and increases energy harvested from the diet (McFall-Ngai et al., 2013). In turn, the host provides sufficient nutrients and suitable living conditions for the microorganisms, and affects the structure and composition of microorganisms in many ways, including genetics, immune status, intestinal environment, and diet (Faith et al., 2011; David et al., 2014). The type of diet and amount of food consumed have been shown to have a considerable impact on the composition of intestinal microbes (Ley et al., 2008a; Muegge et al., 2011). Although host diet provides the major source of substrates that support microbial growth, microbes can also use host-derived substrates, including mucus glycans, nutrients in sloughed epithelial cells, and biliary secretions (Leser et al., 2000; Hooper et al., 2002; Sonnenburg et al., 2005). When the host is fasting, however, there are few or no dietary substrates available for intestinal microbes. Prolonging the fasting period of the host may result in the selective development of gut microbes toward microbial communities capable of decomposing host-derived substrates (e.g., mucin).

Hibernation is a typical example of extended fasting, and is a key feature in the biology of many hibernating mammals (Lyman et al., 1982; Carey et al., 2003; Florant and Healy, 2012). In these species, excessive diets lead to a large accumulation of fat stores in the summer and early fall, followed by voluntary fasting, which can last for up to 8 months. Despite this, these animals manage to maintain the integrity of their organ systems and protect their bodies from damage. Therefore, hibernating mammals provide a unique, natural model system for studying the effects of dietary changes on gut microbiota and host-microbiota symbiosis. Previous studies have shown that hibernation changes the structure and function of intestinal microbes (Carey et al., 2013; Dill-McFarland et al., 2014, 2016). Phylum-level changes in gut microbiota composition over the hibernation cycle are common in herbivores (e.g., small rodents, such as Arctic ground squirrels, thirteen-line ground squirrels) and in carnivores (e.g., large mammals, such as brown bears) (Carey et al., 2013; Dill-McFarland et al., 2014). In these studies, the relative abundance of *Firmicutes* decreased, while *Bacteroidetes* increased, in both animals; however, the relative abundance of *Verrucomicrobia* decreased in brown bears and increased in ground squirrels (Carey et al., 2013; Dill-McFarland et al., 2014, 2016). The taxonomic shifts in the microbiotas of ground squirrels correlate well with known substrate preferences of some of the major taxa of the mammalian gut. The structure of the gut microflora in winter is mainly composed of the *Verrucomicrobia* taxa (Derrien et al., 2004), which can decompose host-derived substrates, or a taxonomic group such as *Bacteroidetes*, which have the ability to switch their complement of carbohydrate-degrading enzymes depending on the availability of dietary vs. host-derived substrates (Sonnenburg et al., 2005). Conversely, the hibernator microbiota is generally depleted in bacteria that prefer dietary polysaccharides, which include many *Firmicutes* species (Flint et al., 2012). Are there similar changes in the intestinal microbes of other hibernating mammals? Answering this question will require studies on more hibernating species, such as insectivores.

Bats, of which a large number of species are seasonal hibernators, are one of the representative groups of hibernating mammals (Whitaker and Rissler, 1992; Geluso, 2007). Unlike the hibernating rodents, which feed on plants, most hibernating bats are insectivores, as well as being small mammals (Burton and Burton, 2002; Carey et al., 2013; Stevenson et al., 2014). An important energy-saving strategy is the decline in metabolic rate during hibernation of many mammals, which plays a vital role in surviving harsh winter environments (Storey and Storey, 2004). The metabolic rate during torpor can be reduced to 4.3% of the basal metabolic rate (Ruf and Geiser, 2015). Moreover, it is commonly known that the major energy source in small mammals switches from carbohydrate to lipid during hibernation (Hampton et al., 2011; Xiao et al., 2015). In addition, studies on the transcriptomes of small hibernating mammals found that the differential expression of genes related to lipid metabolism in hibernating bats is consistent with other small mammals (Williams et al., 2005; Xiao et al., 2015). However, it is unclear whether the changes in gut microbiota are similar in bats and other small hibernating mammals. Furthermore, recent studies of the gastrointestinal flora in bats have mainly focused on the effects of different types of diet (Phillips et al., 2012). Maliničová et al. (2017) utilized traditional culture methods for a dynamic study of the effects of hibernation on the intestinal microbes of bats; however, their results were limited because most intestinal bacteria cannot be cultivated. Using new high-throughput sequencing technologies, such as 16S ribosomal RNA (rRNA) amplicon sequencing, we expect that far more information on the effects of hibernation on intestinal microbiota can be obtained compared with traditional methods. Importantly, seasonal changes in the intestinal microbes of hibernating bats have not been studied.

The greater horseshoe bat (*Rhinolophus ferrumequinum*), a typical hibernating insectivore, is widely distributed in Europe, Africa, South Asia, Australia, and China (Burton and Burton, 2002). In China, its habitat ranges from the northeastern to southwestern regions (Wang, 2003). During hibernation, *R. ferrumequinum* enters a state of torpor, with a lower body temperature (T_b_) of about 10°C, and spontaneous interbout arousals (IBAs) (Park and Ransome, 2000; Burton and Burton, 2002). It has become a model species in hibernation studies of bats. In this study, 16S rRNA amplicon sequencing was used to investigate the effects of hibernation on the intestinal microbes of *R. ferrumequinum*, and PICRUSt was applied to generate a simple prediction of functional gut microbiota. The results of this study enhance our understanding of the symbiotic and adaptive evolution of hibernating mammals and their intestinal microbes.

## Materials and Methods

### Animals

All bats were collected from an artificial canal in Jiyuan City, Henan Province, between September 2017 and July 2018. There were five collection time points over the annual hibernation cycle (Table 1): early spring (*n* = 8; mid-April, AA); early summer (*n* = 7; end of July, JA); late summer (*n* = 8; mid-September, SA); early winter-torpor (*n* = 8; end of January, JT); and early winter-IBA (*n* = 8; end of January, JIBA).

**TABLE 1:** Summary of sample collection information for the study bats.

**Parameter**	**Groups**				
	**Early summer (*n* = 7)**	**Late summer (*n* = 8)**	**Torpor (*n* = 8)**	**IBA (*n* = 8)**	**Early spring (*n* = 8)**
Forearm length	60.74 ± 1.81a	60.84 ± 1.63a	60.97 ± 1.23a	60.32 ± 1.43a	60.35 ± 1.37a
Weight	21.8 ± 1.84a	17.62 ± 0.94b	22.15 ± 1.71a	24.00 ± 1.52a	18.01 ± 1.86b
Body temperature	38.4 ± 1.17a	35.57 ± 1.76a	11.87 ± 4.32b	30.07 ± 5.17a	33.33 ± 5.03a

*Values shown are means ± SE. Different letters (a, b) indicate significant differences between the sample periods (*P* ≤ 0.05).*

During active seasons, bats were collected using mist nets and taken back to a temporary laboratory in the field, where sampling processes immediately followed. During the hibernating season, bats were collected from a cave wall using hand nets and then taken back to the temporary laboratory in the field. Eight active bats were immediately sacrificed to constitute the IBA group. For the torpor group, another eight bats were placed in a small refrigerator at about 10°C to mimic the environmental temperature and allow them to re-enter torpor (∼24 h). To eliminate the effects of age, all of the bats collected for this study were adults. Age was estimated according to the degree of ossification of the epiphyseal spacing (Kunz and Anthony, 1982).

### Sample Collection

Before sampling, we measured the weight and forearm length of the bats (Table 1). Bats were then sacrificed by cervical dislocation and immediate decapitation. Body temperature (T_b_) was promptly measured and recorded (Table 1) by insertion of a thermal probe into the body cavity. The intact guts were rapidly removed for collection of intestinal contents, which were immediately frozen in liquid nitrogen and stored at −80°C until DNA extraction.

Sample collection was carried out with the permission of the local forestry department. All studies in this experiment were approved by the National Animal Research Authority of Northeast Normal University, China (approval number: NENU-20080416).

### DNA Extraction

The intestinal contents of each sample were homogenized. We then used 0.1 g per sample for the extraction of microbial genomic DNA using the DNeasy PowerWater kits (QIAGEN, Inc., Netherlands), following the manufacturer’s instructions. DNA samples were stored at −80°C until further analysis. The quantity of extracted DNA was measured using a NanoDrop ND-1000 spectrophotometer (Thermo Fisher Scientific, Waltham, MA, United States) and the quality was assessed using agarose gel electrophoresis.

### 16S rDNA Amplicon Pyrosequencing

PCR amplification of the V3–V4 region of bacterial 16S rRNA genes was performed using forward primer 308F (5′-ACTCCTACGGGAGGCAGCA-3′) and reverse primer 806R (5′-GGACTACHVGGGTWTCTAAT-3′) (Dennis et al., 2013). Sample-specific 7-bp barcodes were incorporated into the primers for multiplex sequencing. A single-step 30-cycle PCR using the HotStarTaq Plus master mix kit (Qiagen, Valencia, CA, United States) was performed under the following conditions: 98°C for 2 min, followed by 25 cycles consisting of denaturation at 98°C for 15 s, annealing at 55°C for 30 s, extension at 72°C for 30 s, and a final elongation step at 72°C for 5 min. PCR amplicons were purified with Agencourt AMPure Beads (Beckman Coulter, Indianapolis, IN, United States) and quantified using the PicoGreen dsDNA Assay Kit (Invitrogen, Carlsbad, CA, United States). After the individual quantification step, amplicons were pooled in equal amounts and pair-end 2 × 300-bp sequencing was performed using the Illlumina MiSeq platform with MiSeq Reagent Kit v3 at Shanghai Personal Biotechnology Co., Ltd. (Shanghai, China).

### Sequence Data Processing

Sequence processing was performed using the QIIME v1.8.0 program (Caporaso et al., 2010). Briefly, raw sequencing reads with exact matches to the barcodes were assigned to respective samples and identified as valid sequences. All sequences were trimmed and de-noised prior to alignment against the Greengenes 16S rRNA gene reference alignment database (DeSantis et al., 2006). Sequences were screened (filter.seqs) and reduced using unique.seqs and pre.cluster. Chimera detection (chimera.uchime) and removal was performed and the remaining high-quality sequences were clustered into operational taxonomic units (OTUs) at 97% sequence identity by UCLUST (Edgar, 2010). A representative sequence was selected from each OTU using default parameters. OTU taxonomic classification was conducted by BLAST searching of the representative sequences set against the Greengenes Database using the best hit (DeSantis et al., 2006).

### Statistical Analysis

Sequence data analyses were mainly performed using QIIME (v1.8.0) and R packages (v3.5.1). OTU-level ranked abundance curves were generated to compare the richness and evenness of OTUs among samples. The Shannon diversity index and Chao1 richness index were calculated using the OTU table in QIIME to determine alpha diversity for the seasonal groups of bats. We then used the rank sum test to calculate significant differences. Beta diversity analysis was performed to investigate structural variation in microbial communities of the different group samples using unweighted and weighted UniFrac distance metrics (Lozupone and Knight, 2005; Lozupone et al., 2007). The significance of differentiation of microbiota structure among groups was assessed by PERMANOVA (Permutational multivariate analysis of variance) using R package “vegan.” Using QIIME, and according to the sample grouping, the unweighted UniFrac distance matrix was used to statistically compare the mean sample distances of different groups. Differences in the Unifrac distances for pairwise comparisons among groups were determined using the Student’s *t*-test and the Monte Carlo permutation test with 1,000 permutations and then visualized through box-and-whisker plots. Taxonomies were grouped at the phylum, class, order, family, and genus levels. Taxa abundances at the phylum, class, order, family and genus levels were statistically compared among groups by Metastats (White et al., 2009). Microbial functions were predicted by PICRUSt (Phylogenetic investigation of communities by reconstruction of unobserved states), based on high-quality sequences (Langille et al., 2013).

## Results

### Microbial Diversity

After quality processing, a total of 1,327,632 16S rRNA gene reads (average 34,041 reads/sample) were obtained, from which 1,750 OTUs were identified to be from gut microbiotas of 39 bats. The rarefaction curve analysis indicated that sequencing captured the majority of microbial diversity in our samples (Supplementary Figure 1).

**FIGURE 1:**
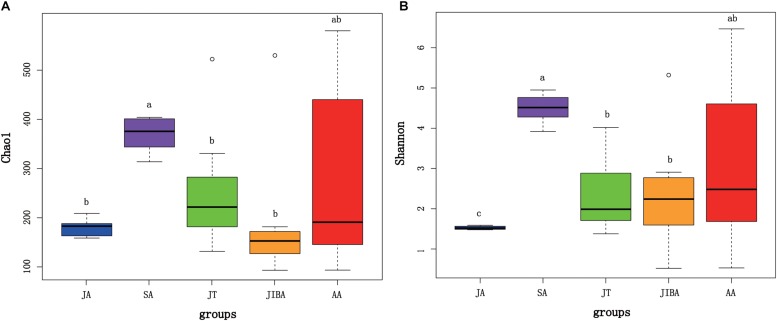
Alpha diversity of microbial communities in the bat gut. Data plotted show the last means ± SE of the Chao1 **(A)** and Shannon **(B)** indexes from rarefaction plots (Supplementary Figure 1). Significant differences in alpha diversity among the groups are indicated by different letters according to Kruskal–Wallis rank sum test (*P* ≤ 0.05). JA, early summer; SA, late summer; JT, early winter-torpor; JIBA, early winter IBA; AA, early spring.

Alpha diversity of gut microbiotas differed significantly among sample periods (Figure 1). The alpha diversity value was highest in the late summer, followed by early spring and early winter, and lowest in the early summer (Figure 1 and Supplementary Figure 1). Alpha diversity (for two indices) was significantly higher in the late summer compared with the early summer (*P* < 0.01) and early winter (*P* < 0.05) (Figure 1). There was no significant difference in the alpha diversity of gut microbiotas between the IBA and torpor groups in early winter. The alpha diversity of gut microbiotas in early winter was not significantly different from that in early spring. However, we found that the Shannon index of gut microbiotas in early summer was significantly lower than those during torpor (*P* = 0.015) and IBA (*P* = 0.037) in early winter (Figure 1B).

### Cluster Analysis of Microbiotas

**FIGURE 2:**
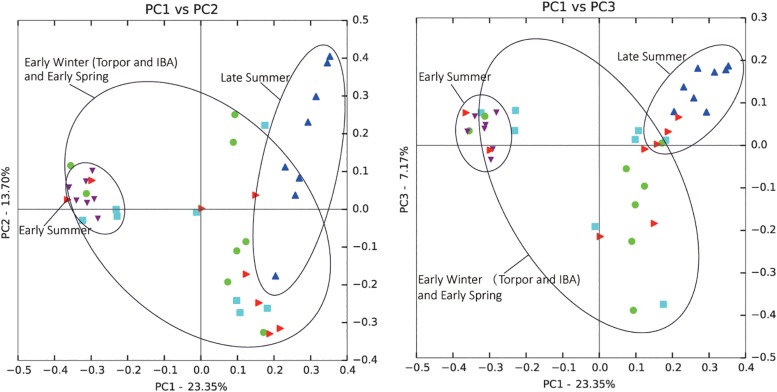
Principal coordinate analysis (PCoA) plots of unweighted UniFrac metrics for bat microbiotas. Each dot represents individual microbiota samples obtained from early spring (red triangles), early summer (purple triangles), late summer (blue triangles), early winter-torpor (light blue squares), and early winter-IBA (green circles). Left, PCA1 vs. PCA2; Right, PCA1 vs. PCA3. Numbers of bats in each seasonal group are shown in Table 1.

**FIGURE 3:**
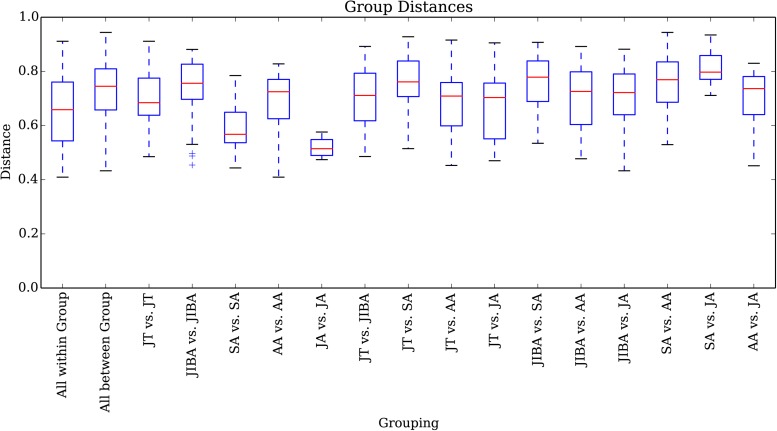
UniFrac distances for pairwise comparisons among groups. The *X*-axis shows pairwise comparisons among groups; the *Y*-axis shows UniFrac distances. The box plot border represents the upper and lower quartile spacing, the horizontal line represents the median value, and the upper and lower tentacles represent the 1.5 × IQR range outside the upper and lower quartiles, respectively. Significant differences in the UniFrac distances for pairwise comparisons among groups are shown in Table 2. If the distance between two groups was significantly greater than that within the groups, the difference between these groups was considered significant.

Comparison of individual bat microbiotas, using principal coordinate analysis (PCoA) based on unweighted UniFrac metric data, showed distinct clustering by season, with most of the variation explained by the first three coordinates (Figure 2). Individuals from early summer and late summer groups clustered separately. Most individuals from early spring and early winter groups (torpor and IBA) clustered together. The thermal and metabolic state of hibernating bats at the time of sampling had little effect on microbiota clustering in either winter group, because individuals in torpor are clustered together with individuals in IBA (Figure 2). The results of PERMANOVA showed significant differences between two groups at least (*P* ≤ 0.001) (Supplementary Table 1). Differences in the UniFrac distances for pairwise comparisons among groups were determined using Student’s *t*-test and the Monte Carlo permutation test with 1,000 permutations to clarify significant differences between two groups. The results showed that the community structure of intestinal microbes in early summer and late summer were significantly different, and that these two groups were also significantly different from other groups. There was no significant difference in the microbial community structure among winter torpor, IBA, and early spring groups (Figure 3 and Table 2).

**TABLE 2:** Results of Student’s *t*-test and the Monte Carlo permutation test of differences in the UniFrac distances for pairwise comparisons among groups.

**Group 1**	**Group 2**	***t* statistic**	***p*-value**
JT vs. JT	JT vs. JIBA	–0.080	1.000
JT vs. JT	JT vs. SA	–2.863	0.708
JT vs. JT	JT vs. AA	0.336	1.000
JT vs. JT	JT vs. JA	0.937	1.000
JIBA vs. JIBA	JIBA vs. SA	–1.273	1.000
JIBA vs. JIBA	JIBA vs. AA	1.025	1.000
JIBA vs. JIBA	JIBA vs. JA	1.128	1.000
JIBA vs. JIBA	JT vs. JIBA	1.031	1.000
SA vs. SA	SA vs. AA	–7.173	0.000^∗∗∗^
SA vs. SA	SA vs. JA	–13.414	0.000^∗∗∗^
SA vs. SA	JIBA vs. SA	–7.922	0.000^∗∗∗^
SA vs. SA	JT vs. SA	–8.717	0.000^∗∗∗^
AA vs. AA	JT vs. AA	0.265	1.000
AA vs. AA	SA vs. AA	–2.211	1.000
AA vs. AA	AA vs. JA	–0.046	1.000
AA vs. AA	JIBA vs. AA	–0.160	1.000
JA vs. JA	JT vs. JA	–5.355	0.000^∗∗∗^
JA vs. JA	JIBA vs. JA	–6.668	0.000^∗∗∗^
JA vs. JA	SA vs. JA	–20.751	0.000^∗∗∗^
JA vs. JA	AA vs. JA	–7.356	0.000^∗∗∗^

*^∗∗∗^*p* value ≤ 0.001.*

### Taxonomic Composition of Bat Microbiotas

**FIGURE 4:**
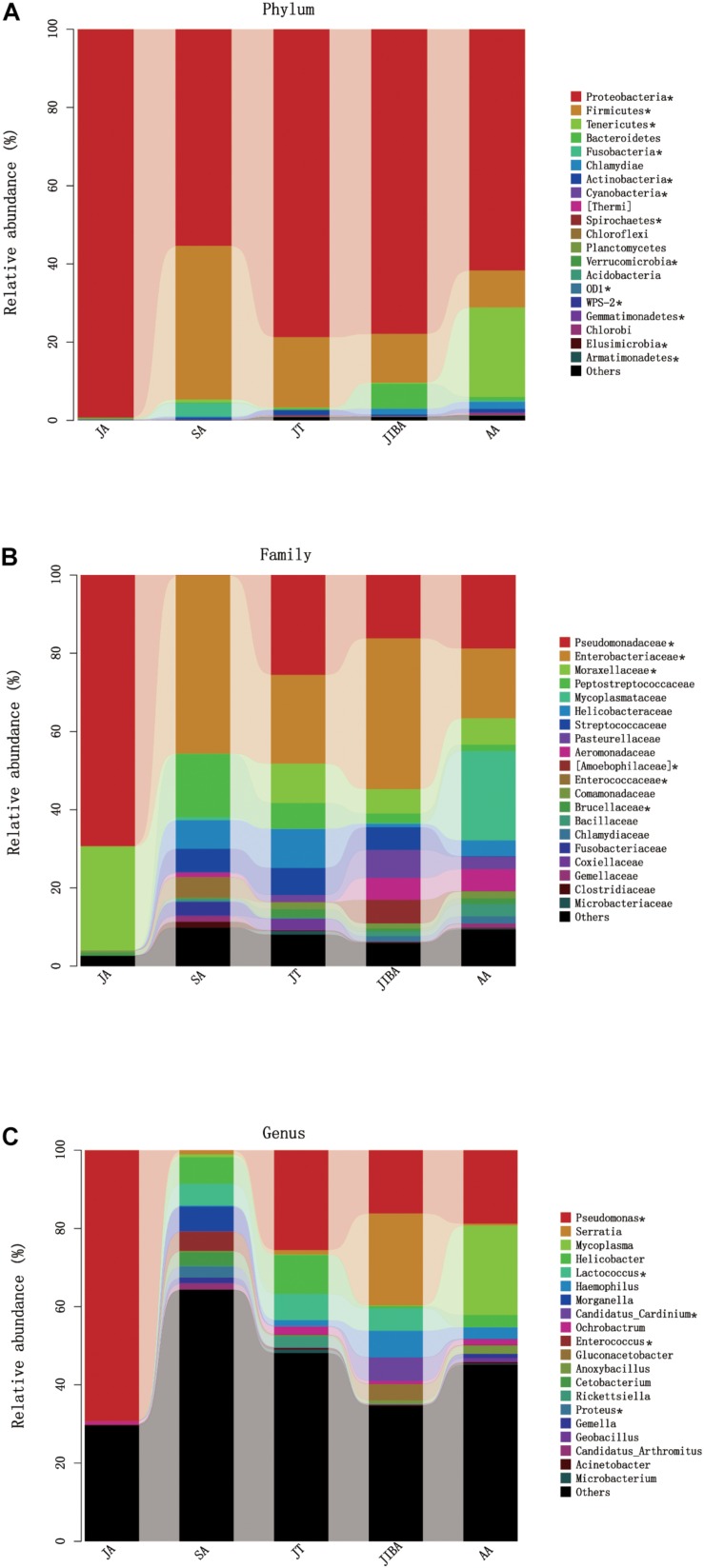
Relative abundance of major taxa in microbiotas of the bat gut. **(A)** Phylum; **(B)** Family; **(C)** Genus. Asterisks represent microbial taxa with significant differences in each seasonal period. The numbers of bats in each seasonal group are shown in Table 1.

Four major bacterial phyla were identified in the *R. ferrumequinum* microbiota, with the majority of sequences classified as *Proteobacteria* (∼74.6%), followed by *Firmicutes* (∼15.9%), *Tenericutes* (∼4.8%), and some *Bacteroidetes* (∼1.6%) (Figure 4 and Supplementary Table 2). The other phyla were *Fusobacteria*, *Chlamydiae*, and *Actinobacteria*, but were not abundant (<1%). An unclassified group of sequences accounted for the remainder of the bacterial diversity of the bat microbiota (Supplementary Table 2).

The majority of *Proteobacteria* sequences identified in the bat microbiota matched the order *Pseudomonadales* and *Enterobacteriates.* The order *Pseudomonadales* were represented primarily by the family *Pseudomonadaceae* and *Moraxellaceae*. At the genus level, dominant groups were *Pseudomonas*. The order *Enterobacteriates* were represented primarily by the family *Enterobacterlaceae*. There were some unclassified *Enterobacteriaceae* and *Serratia*, and less abundant *Proteus* at the genus level (Supplementary Table 2). *Firmicutes* were dominated by *Lactobacillales* and *Clostridiales*. *Lactobacillus* were represented by *Streptococcaceae*, *Enterococcaceae*, and some unclassified *Lactobacillales*, with most OTUs matching to the genera *Lactococcus* and *Enterococcus*. *Clostridiales* was mainly represented by *Peptostreptococcaceae*, which contained some taxa of the unclassified *Peptostreptococcaceae*. The sequences of *Tenericutes* mainly matched the order *Mycoplasmatales*, which was primarily represented by the family *Mycoplasmataceae*. The taxonomic group at the genus level was *Mycoplasma* (Figure 4 and Supplementary Table 2).

At the phylum level, the relative abundance of *Proteobacteria* was highest in early summer and significantly lower in other periods. The relative abundance of *Firmicutes* was lowest in early summer and significantly increased in late summer, followed by significant decreases in subsequent periods. The relative abundance of *Tenericutes* was significantly higher in early spring than in other periods and decreased to undetectable levels in early summer (Figure 4 and Table 3). At the family level, the relative abundance of *Enterobacteriaceae* significantly increased from early summer to early winter, and the IBA group had double that of the torpor period (Figure 4 and Table 3). The changes in the relative abundance of *Pseudomonadaceae* and *Moraxellaceae* were consistent, with significantly higher values in early summer than in other periods, the lowest being in late summer, and followed by significant increases in subsequent periods. *Enterococcaceae* was significantly higher in late summer than in other periods, reduced in early winter, and reduced to undetectable levels in early summer (Figure 4 and Table 3). At the genus level, *Pseudomonas* was obviously affected by season; the relative abundance was highest in the early summer group and reduced to the lowest abundance in the late summer group, then increased during hibernation, but was still significantly lower than in early summer. The relative abundances of *Enterococcus*, *Lactococcus*, and *Proteus* were highest in late summer, reduced in early winter, and almost undetectable in early summer. Seasonal changes in diet also influenced the relative abundance of some of the less common taxa (Figure 4 and Table 3).

**TABLE 3:** Relative abundances of taxa with seasonal differences in bat gut microbiotas.

		**Early summer (*n* = 7)**	**Late summer (*n* = 8)**	**Torpor (*n* = 8)**	**IBA (*n* = 8)**	**Early spring (*n* = 8)**
Phylum	Proteobacteria	99.28 ± 0.27a	55.36 ± 24.70b	79.05 ± 29.46b	77.88 ± 19.74b	61.63 ± 32.95b
	Firmicutes	0.10 ± 0.07c	39.28 ± 19.62a	18.03 ± 10.60b	12.46 ± 6.62b	9.98 ± 14.83b
	Tenericutes	0.00 ± 0.00c	0.88 ± 0.38b	0.17 ± 0.10b	0.32 ± 0.15b	22.90 ± 13.32a
	Bacteroidetes	0.37 ± 0.00a	0.20 ± 0.00a	0.42 ± 0.00a	6.30 ± 0.02a	0.91 ± 0.00a
	Verrucomicrobia	0.02 ± 0.00a	0.00 ± 0.00b	0.02 ± 0.00a	0.04 ± 0.00a	0.03 ± 0.00a
Family	Enterobacteriaceae	0.03 ± 0.01c	48.63 ± 10.52a	23.94 ± 14.58b	40.47 ± 12.09a	18.74 ± 8.28b
	Pseudomonadaceae	70.50 ± 0.22a	0.01 ± 0.01c	26.04 ± 12.66b	16.47 ± 10.80b	19.27 ± 11.26b
	[Amoebophilaceae]	0.00 ± 0.00b	0.00 ± 0.00b	0.02 ± 0.02b	6.06 ± 0.60a	0.01 ± 0.00b
	Moraxellaceae	27.10 ± 0.15a	0.08 ± 0.04c	10.29 ± 4.76b	6.39 ± 3.96b	6.96 ± 4.12b
	Enterococcaceae	0.00 ± 0.00c	5.62 ± 1.89a	0.10 ± 0.08b	0.02 ± 0.01b	0.10 ± 0.00b
	Brucellaceae	0.85 ± 0.06ab	0.11 ± 0.07b	2.16 ± 1.02a	0.85 ± 0.4ab	1.61 ± 1.02ab
	Desulfovibrionaceae	0.00 ± 0.00b	0.00 ± 0.00b	0.00 ± 0.00b	0.00 ± 0.00b	0.53 ± 0.52a
	Brevinemataceae	0.00 ± 0.00b	0.00 ± 0.00b	0.18 ± 0.01a	0.00 ± 0.00b	0.01 ± 0.00b
	Chitinophagaceae	0.27 ± 0.02a	0.07 ± 0.04b	2.95 ± 0.1a	0.11 ± 0.03b	0.18 ± 0.06b
	Pseudonocardiaceae	0.45 ± 0.07a	0.08 ± 0.00b	0.14 ± 0.06a	0.00 ± 0.00.c	0.16 ± 0.10a
	Caulobacteraceae	2.50 ± 0.01a	0.03 ± 0.02b	0.24 ± 0.07ab	0.12 ± 0.05b	0.23 ± 0.09ab
Genus	*Pseudomonas*	97.47 ± 0.22a	0.03 ± 0.03c	36.24 ± 21.742b	22.13 ± 14.56b	26.62 ± 15.58b
	*Candidatus_Cardinium*	0.00 ± 0.00b	0.00 ± 0.00b	0.01 ± 0.01b	6.33 ± 6.32a	0.01 ± 0.01b
	*Lactococcus*	0.00 ± 0.00b	14.01 ± 0.04a	9.57 ± 0.08ab	7.23 ± 7.17ab	0.14 ± 0.11b
	*Enterococcus*	0.00 ± 0.00b	13.62 ± 4.33a	0.27 ± 0.22b	0.08 ± 0.04b	0.18 ± 0.00b
	*Proteus*	0.00 ± 0.00b	7.34 ± 2.41a	0.00 ± 0.00b	0.00 ± 0.00b	0.00 ± 0.00b
	*Brevinema*	0.00 ± 0.00b	0.00 ± 0.00b	1.33 ± 0.01a	0.00 ± 0.00b	0.20 ± 0.20ab
	*Sediminibacterium*	0.37 ± 0.02a	0.34 ± 0.25a	0.53 ± 0.16a	0.15 ± 0.04b	0.49 ± 0.20a

*Values shown are means ± SE. Different letters (a, b, c) indicate significant differences (*p* value < 0.05, Metastats). Sample sizes (*n*) are in parentheses.*

### Significant Seasonal Changes in Gut Microbial Functions Predicted via PICRUSt

**FIGURE 5:**
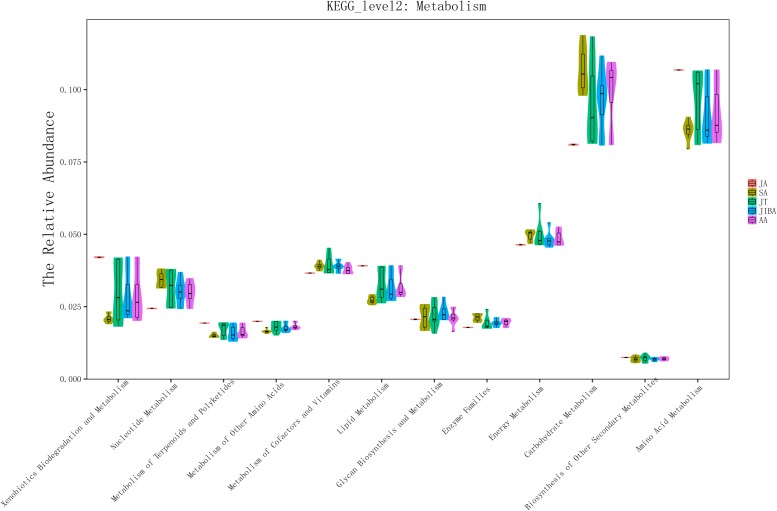
16S rRNA gene sequencing of bat gut microbiota samples analyzed using PICRUSt. The abscissa shows all of the metabolic-related functional groups of the KEGG second level. The relative abundance of all functional taxa had significant differences between the groups for at least two periods (Kruskal–Wallis’s test, *p* < 0.01), except for Biosynthesis of Other Secondary Metabolites and Glycan Biosynthesis and Metabolism (Supplementary Table 3).

We used PICRUSt to perform functional predictions on the results of 16S rDNA sequencing. Based on the KEGG pathway database, analysis of the second level of functional categories of the KEGG pathway suggested that the relative abundance of most predicted functional categories changed significantly among the different groups (Kruskal–Wallis test, *P* < 0.05) (Supplementary Table 3), including metabolism of lipids, carbohydrates, energy, and amino acids (Figure 5 and Supplementary Table 3). The relative abundance of functional categories related to lipid metabolism was highest in early summer and lowest in late summer, and significantly increased in early winter and early spring compared with late summer, but was still significantly lower than that in early summer. The relative abundance of carbohydrate-related functional categories was lowest in the early summer and significantly increased in the late summer, followed by a significant decrease in torpor in the early winter. The relative abundance of energy metabolism was lowest in early summer, and in late summer and early winter torpor increased significantly. The relative abundance of functional categories associated with amino acid metabolism was significantly higher in early summer than in other periods (Figure 5 and Supplementary Table 3). These results indicated that the metabolic function of gut microbes in hibernating bats are altered the most by the changes in season.

## Discussion

Like other fat-storing hibernators, *R. ferrumequinum* stores large amounts of adipose tissue during active seasons, uses stored lipids as the main source of energy during hibernation, and begins refeeding in spring (Park and Ransome, 2000; Xiao et al., 2015). During hibernation, the T_b_ drops to about 10°C (slightly higher than the ambient temperature) with spontaneous awakening during hibernation (Park and Ransome, 2000). During IBA periods, the T_b_ returns to the active level of about 34–38°C. This hibernation-related physiological change causes a great alteration in the intestinal environment of bats, which greatly impacts the intestinal microbes. In this investigation, the microbial diversity, composition, and function of the intestines of *R. ferrumequinum* were each found to undergo seasonal changes.

### Seasonal Changes in Diet Is the Main Factor Driving Intestinal Microbial Variation

The diversity in gut microbes gradually decreased sequentially: in the order of late summer, early spring, early winter and early summer, which was consistent with the seasonal changes in the diet of *R. ferrumequinum*. It has been reported that their diet is most diverse in autumn (Jones, 1990). Thus, the higher diversity of gut microbes in late summer may be associated with a more diverse diet at that time, which is close to autumn. Effects of hibernation were also evident in the gut microbial diversity, which was lower during hibernation than in early spring and late summer. This may be due to the bats fasting during hibernation. These changes in diversity are consistent with the findings in brown bears and ground squirrels (Carey et al., 2013; Stevenson et al., 2014; Sommer et al., 2016). Similarly to the results in ground squirrels (Carey et al., 2013), the diversity of bat gut microbes in early summer was lower than that in early winter, which suggested that the intestinal microbes become more stable and less diverse in the summer, as the time of re-feeding increases. The summer period may be dominated by species that have a competitive advantage in the presence of abundant and continuously available dietary substrates.

The PCoA based on the unweighted UniFrac metric showed that the community structure of bat gut microbes significantly clustered by seasons, with significant differences between samples from early summer and late summer. *R. ferrumequinum* mainly feeds on *Lepidopteran* and *Coleopteran* insects, and there are significant seasonal changes in food composition (Jones, 1990; Wang et al., 2010). Studies have shown that the proportions of *Lepidoptera* in the diet are higher in the early summer, while in the late summer, the proportion of *Coleoptera* is larger (Wang et al., 2010). Changes in food composition may be the main reason for the separate clustering of intestinal microflora in early summer and late summer. Most individuals from early spring and early winter (torpor and IBA) clustered together. The individuals in the torpor group clustered with the individuals in the IBA group, indicating that the euthermic and metabolic status of the hibernating bats during sampling had little effect on the microbial clustering of the winter group, which is consistent with a study of the ground squirrel (Stevenson et al., 2014). In the early winter hibernation period, bats have fasted, so that the early winter-torpor and IBA groups may have been dominated by some microbial communities capable of decomposing host-derived substrates. The individuals in early spring and early winter clustered, indicating that the microbial community structure changed slightly during the spring. That may have been because the bats were just starting to awaken from hibernation. During early spring, when some individuals may eat a little food, the gut microbe diversity was higher than that in winter, although not significantly. This observation indicated that the diversity of the bats’ gut microbiota was recovering. Ecosystem function is often positively correlated with species richness (Hooper et al., 2005; Maestre et al., 2012; Cardinale et al., 2013; Wagg et al., 2014). Significantly decreased gut microbial diversity during early winter indicates that hibernation, accompanied by slower metabolism, may weaken microbial ecosystem function in the gastrointestinal tract.

### Changes in Intestinal Microbes of Hibernating Bats Are the Same as Those of Other Small Hibernating Mammals

Our taxonomic composition analysis across different seasonal periods found that, unlike other mammals, the gut microbiota of *R. ferrumequinum* was dominated by the *Proteobacteria*, while *Firmicutes*, *Tenericutes*, and *Bacteroidetes* occupied smaller parts. *Proteobacteria* was also found to be the primary bacteria in other studies on the microbial composition of the bat gut (Carrillo-Araujo et al., 2015; Nishida and Ochman, 2017; Hughes et al., 2018; Ingala et al., 2018). In other mammals, such as ground squirrels and brown bears, as well as humans, *Bacteroides* and *Firmicutes* dominate, while the *Proteobacteria* account for only a small part of the microbiota. In the human intestinal tract, *Proteobacteria* comprise less than 5% of the total (Shin et al., 2015). Therefore, our data indicate that the dominant species of gut microbiota differs between bats and other hibernating mammals.

In our comparison of the composition of bat gut microbes at different seasonal times, we found significant differences in the relative abundance of major bacterial phyla. The relative abundance of *Proteobacteria* was higher in early winter than in late summer, the relative abundance of *Firmicutes* was significantly lower in early winter than in late summer, and the relative abundance of *Tenericutes* was significantly higher in the early spring than in other periods. In the study of ground squirrels, the relative abundance of *Firmicutes* decreased, while *Bacteroidetes* and *Verrucomicrobia* increased during hibernation (Carey et al., 2013; Stevenson et al., 2014). In brown bears, the relative abundance of *Firmicutes* decreased and *Bacteroides* increased during hibernation, but the relative abundance of *Verrucomicrobia* decreased (Sommer et al., 2016). Although the dominant intestinal microbe differed between the bats in our study and some other mammals, there were similar changes in the gut microbiota of bats and ground squirrels. During hibernation in our bats, the relative abundance of *Firmicutes* decreased, and the relative abundance of *Bacteroidetes* and *Verrucomicrobia* increased, although the changes in *Bacteroidetes* were not significant. These results suggested that the composition of the gut microbiome of small mammals, including bats and squirrels, tend to be similarly altered in response to hibernation.

### Seasonal Changes in the Metabolic Function of Bat Intestinal Microbes

The relative abundances of most of the predicted functional taxa of bat gut microbials in the KEGG pathway changed significantly by season. The primary changes occurred in the relative abundance of metabolism-related functional taxa. In seasonal comparisons of functional categories, those associated with carbohydrate metabolism had significantly lower relative abundances in early winter-torpor compared with late summer, while the reverse was true for those categories associated with lipid metabolism. These results suggested that the metabolic function of the gut microflora is converted from carbohydrate-related to lipid-related functional categories during hibernation. This may be related to the transformation in substrate utilization involved in energy metabolism during hibernation, which involves an important physiological shift from carbohydrate oxidation to lipid catabolism (Carey et al., 2003). Studies of transcriptomes in small hibernating mammals have revealed that many genes involved in metabolism are differentially expressed during hibernation. Specifically, the genes involved in carbohydrate catabolism are downregulated during hibernation, while genes responsible for lipid catabolism are upregulated (Carey et al., 2013; Stevenson et al., 2014; Han et al., 2015; Xiao et al., 2015; Sommer et al., 2016). We also found that the relative abundance of functional categories associated with amino acid metabolism increased in the early winter compared with the late summer, consistent with other studies showing that hibernating bats have elevated levels of amino acid metabolism during hibernation (Pan et al., 2013). We propose that such an increase in amino acid metabolism may produce the very metabolic substrates that are required to alter the relative abundance of functional categories of gut microbes involved in amino acid metabolism. Overall, such results are consistent with seasonal changes in intestinal microbial metabolic function that support the co-evolution of gut microbes and hibernating hosts. Studies of ground squirrels and brown bears also found that intestinal microbes can regulate metabolic function (Carey et al., 2013; Stevenson et al., 2014; Sommer et al., 2016). Seasonal differences in gut microbes may contribute to the metabolic changes that occur in hibernating animals. The interaction between the host and its gut microbes appears to be vital in promoting the adaptation of the hibernating host to the extreme environment of fasting in winter.

### Seasonal Variation in Intestinal Microbes and Adaptation to Extreme Environments in Bats

In this study, we also predicted the functions of some microbial species. For example, the relative abundance of *Firmicutes* was highest in late summer, which may be associated with fattening before entering hibernation. Some studies on non-hibernating mammals have suggested that changes in the relative abundance of the *Firmicutes* phylum are associated with fat accumulation and the potential for obesity (Ley et al., 2008a; Turnbaugh and Gordon, 2009), although not all studies support this opinion (Duncan et al., 2007; Schwiertz et al., 2010). In our study, the weight of bats in late summer was significantly lower than that in early summer and early winter, and not significantly different from that in spring, indicating that our late-summer sampling occurred before the fattening period. Indeed, the relative abundance of *Firmicutes* increased sharply before the bats entered the fattening period in autumn and decreased after entering hibernation. In addition, the relative abundance of *Pseudomonas* was significantly increased during hibernation. Bacteria of this genus are able to grow at a wide range of temperatures, from 5° to 42°C (Termine and Michel, 2009). Moreover, fasting hosts use lipids as the main energy source during hibernation, and most species of *Pseudomonas* secrete a low-temperature lipase that can break down lipids. The optimum temperature for the low-temperature lipase is 7–10°C, and it also has certain catalytic activity at 0°C (Roh and Villatte, 2008; Ji et al., 2015; Jain et al., 2017). The low T_b_ and shift in energy source from glucose to lipids in hibernating bats may be responsible for the significant increase in the relative abundance of *Pseudomonas* during hibernation. Studies in *Rhinolophus euryale* have shown that *Pseudomonas* is also predominant in the stool samples of hibernating *R. euryale* (Maliničová et al., 2017). Our study revealed that the gut microbiomes of bats and ground squirrels tend to have similar changes in response to hibernation. In a study of hibernating ground squirrels, it was shown that hibernation increased the relative abundance of *Bacteroidetes* and *Verrucomicrobia* (phyla which contain species capable of surviving on host-derived substrates such as mucins), and reduced the relative abundance of *Firmicutes* (many of which prefer dietary polysaccharides). The ground squirrel microbiota is restructured each year in a manner that reflects differences in the abilities of different bacterial taxa to survive in the changing environment of the hibernator gut (Carey et al., 2013). Therefore, seasonal changes in the intestinal microbial composition of hibernating bats may reflect compatibility with microbial functions. Seasonal changes in diet alter the structure of the gut microbiota; such rebuilding of microbial communities in the bat gut may alter the interactions between microbial species and the overall function of the ecosystem. These may be important factors for host adaptation to extreme winter environments.

## Conclusion

Seasonal changes occurred in the diversity and composition of the intestinal microbes of hibernating *R. ferrumequinum*. The alpha diversity value was highest in the late summer, followed by early spring and early winter, and lowest in the early summer. Seasonal changes in diet strongly affected taxonomic representation of the dominant phyla. The relative abundance of *Proteobacteria* was highest in the early summer and significantly lower in other periods. The relative abundance of *Firmicutes* was lowest in the early summer and significantly increased in the late summer, followed by a significant decrease in the early winter and early spring. Comparison of changes in gut microbiota between bats and other hibernating mammals suggested that gut microbiota tend to show similar responses to hibernation in some small mammals (i.e., bats and ground squirrels). The results of functional prediction by PICRUSt showed that the relative abundance of metabolism-related pathways changed in different seasons. This indicates the important role of gut microbes in the adaptation of hibernating animals to the extreme environment of fasting in winter. More work is needed to understand the functions of gut microorganisms in hibernating mammals during different periods. For example, we plan to investigate whether seasonal changes in the intestinal microbiota of hibernating mammals reprograms the metabolic functions of the microorganisms using a combination of metagenomics and metabolomics.

## Data Availability Statement

The datasets generated for this study can be accessed from NCBI Sequence Read Archive (SRA), SRR8756041–SRR8756079.

## Ethics Statement

According to the regulations of Wildlife Conservation of the People’s Republic of China (Chairman Decree [2004] No. 24), permits are required only for species included in the list of state-protected and region-protected wildlife species. *R. ferrumequinum* is not an endangered or region-protected animal species, so no specific permission was required. Sampling was conducted outside the protected areas, with permission of the local forestry department. All experimental procedures carried out in this study were approved by the Forestry Bureau of Jilin Province of China (approval number: [2006]178). And all efforts were made to minimize suffering of animals in the process of collecting samples.

## Author Contributions

YX and GX contributed to the design of the study. GX, YZ, and SL collected the gut content samples in all period. YX collected samples in late summer. AL and ZL collected samples in winter. GX analyzed the data. GX, YX, SL, HZ, and JF contributed to the writing of the manuscript.

## Conflict of Interest

The authors declare that the research was conducted in the absence of any commercial or financial relationships that could be construed as a potential conflict of interest.
