# Supplementary material for: Seasonal Changes in Gut Microbiota Diversity and Composition in the Greater Horseshoe Bat
Source: Front Microbiol. 2019 Oct 1;10:2247. doi: 10.3389/fmicb.2019.02247 (PMC6779692; doi:10.3389/fmicb.2019.02247)
Supplement: Supplementary file 1 [file Table_1.DOCX]

Supplementary Material

# Supplementary Figures and Tables

## Supplementary Figures

**
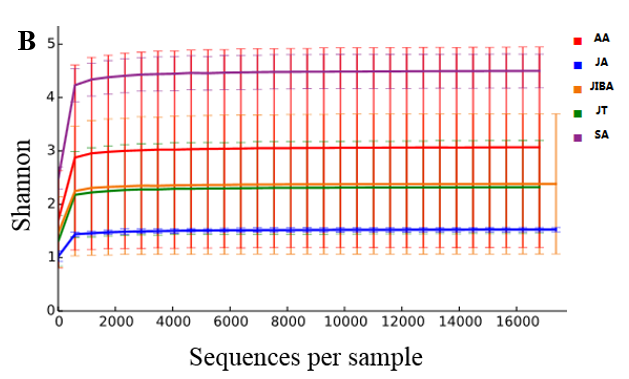

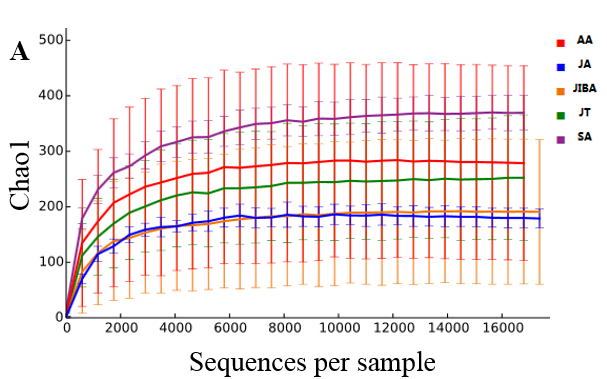
Supplementary Figure 1**

**Supplementary Figure 1.** **Rarefaction curves of gut microbial communities.** A) Chao1 metric, B) Shannon metric. Each line represents a sampling period, and each period is the mean +/- standard error. Symbols are: Early Spring (AA, red; n=8), Early Summer (JA, blue; n=7), Late Summer (SA, purple; n=8), Early Winter torpor (JT, green; n=8) and Early Winter IBA (JIBA, orange; n=8). Rarefaction plots were produced in QIIME 1.6.0.

## Supplementary Tables

**Supplementary Table1 A)** Results of Unweighted UniFrac PERMANOVA analysis

|  | Df | Sums Of Sqs | MeanSqs | F.Mode1 | R2 | P＞(Fr) |
| --- | --- | --- | --- | --- | --- | --- |
| temp[[opts$category]] | 4 | 2.4572 | 0.61431 | 2.7924 | 0.24728 | 0.001*** |
| Residuals | 34 | 7.4798 | 0.21999 |  | 0.75272 |  |
| Total | 38 | 9.9370 |  |  | 1.00000 |  |

****P*-value≤0.001.

**B)** Results of weighted UniFrac PERMANOVA analysis

|  | Df | Sums Of Sqs | MeanSqs | F.Mode1 | R2 | P＞(Fr) |
| --- | --- | --- | --- | --- | --- | --- |
| temp[[opts$category]] | 4 | 0.9030 | 0.225760 | 3.0936 | 0.26684 | 0.001*** |
| Residuals | 34 | 2.4812 | 0.072976 |  | 0.73316 |  |
| Total | 38 | 3.3842 |  |  | 1.00000 |  |

****P*-value≤0.001.

**Supplementary** **Table2** Microbial taxa in Rhinolophus ferrumequinum gut contents

| Phylum | Class | Order | Family | Genus |
| --- | --- | --- | --- | --- |
| Proteobacteria | Gamaproteobacteria | Pseudomonadales | Pseudomonadaceae | Pseudomonas |
|  | Gamaproteobacteria | Pseudomonadales | Moraxellaceae | * |
|  | Gamaproteobacteria | Enterobacteriates | Enterobacterlaceae | * |
|  | Gamaproteobacteria | Enterobacteriates | Enterobacterlaceae | Serratia |
|  | Gamaproteobacteria | Enterobacteriates | Enterobacterlaceae | Morganella |
|  | Gamaproteobacteria | Enterobacteriates | Enterobacterlaceae | Gluconacetobacter |
|  | Gamaproteobacteria | Enterobacteriates | Enterobacterlaceae | proteus |
|  | Gamaproteobacteria | Pasteurellales | Pasteurellaceae | Haemophilus |
|  | Gamaproteobacteria | Aeromonadales | Aeromonadaceae | * |
|  | Gamaproteobacteria | Legionellales | Coxiellaceae | Rickettsiella |
|  | Epsiconproteobacteria | Gampylobacterales | Helicobacteraceae | Helicobacter |
|  | Betaproteobacteria | Burkholderiales | Comamonadaceae | * |
|  | Alphaproteobacteria | Rhizobiales | Brucellaceae | Ochrobactrum |
| Firmicutes | Bacilli | lactobacillales | Streptococcaceae | Lactococcus |
|  | Bacilli | lactobacillales | * | * |
|  | Bacilli | lactobacillales | Enterococcaceae | Enterococcus |
|  | Bacilli | Bacillales | Bacillaceae | Anoxybacillus |
|  | Clostridia | Clostridiales | Peptostreptococcaceae | * |
| Tenericutes | Mollicutes | Mycoplasmatales | Mycoplasmataceae | Mycoplasma |
| Bacteroidetes | Cytophagia | Cytophagales | Amoebophilaceae | Candidatus Cardinium |
| Fusobacteria | Fusobacteriia | Fusobacteriales | Fusobacteriaceae | Cetobacterium |
| Chlamydiae | Chlamydiia | Chlamydiales | Chlamydiaceae | * |
| Actinobacteria | Actinobacteria | Actinomycetales | Microbacteriaceae | Microbacterium |

Asterisks indicate unclassified members of higher level taxa

**Supplementary Table3** PICRUSt showing predicted relative abundance of all KEGG pathway (Level 2 KOs)

| KEGG pathways | Early Summer | Late Summer | Early Winter | | Early Spring |
| --- | --- | --- | --- | --- | --- |
|  |  |  | Torpor | IBA |  |
| Amino Acid Metabolism | 10.68±0.00a | 8.59±0.30b | 9.67±1.04b | 9.09±0.92b | 9.16±0.92b |
| Biosynthesis of Other Secondary Metabolites | 0.75±0.00a | 0.69±0.07a | 0.71±0.10a | 0.68±0.04a | 0.70±0.05a |
| Carbohydrate Metabolism | 8.10±0.01c | 10.69±0.71a | 9.46±1.30b | 9.66±0.097ab | 9.95±1.05ab |
| Energy Metabolism | 4.64±0.00b | 4.97±0.16a | 4.98±0.45a | 4.81±0.24ab | 4.85±0.24ab |
| Enzyme Families | 1.78±0.00c | 2.13±0.12a | 1.93±0.20b | 1.93±0.10b | 1.95±0.10b |
| Glycan Biosynthesis and Metabolism | 2.06±0.01a | 2.12±0.35a | 2.19±0.39a | 2.29±0.25a | 2.13±0.25a |
| Lipid Metabolism | 3.91±0.00a | 2.73±0.11c | 3.26±0.51b | 3.14±0.44b | 3.19±0.40b |
| Metabolism of Cofactors and Vitamins | 3.66±0.00b | 3.90±0.11a | 3.94±0.30a | 3.90±0.16a | 3.78±0.14ab |
| Metabolism of Other Amino Acids | 1.99±0.00a | 1.65±0.05c | 1.80±0.16b | 1.76±0.13b | 1.82±0.09b |
| Metabolism of Terpenoids and Polyketides | 1.93±0.00a | 1.50±0.06b | 1.72±0.23b | 1.58±0.22b | 1.62±0.19b |
| Nucleotide Metabolism | 2.44±0.00c | 3.44±0.25a | 3.15±0.58ab | 3.01±0.39b | 2.96±0.34b |
| Xenobiotics Biodegradation and Metabolism | 4.20±0.01a | 2.09±0.14b | 2.99±0.98b | 2.80±0.79b | 2.85±0.80b |
| Metabolic Diseases | 0.06±0.00c | 0.08±0.00a | 0.08±0.01ab | 0.07±0.01ab | 0.07±0.00b |
| Cancer | 0.12±0.00a | 0.09±0.01b | 0.11±0.01b | 0.10±0.01b | 0.11±0.00b |
| Neurodegenerative Diseases | 0.36±0.00a | 0.09±0.01b | 0.30±0.14ab | 0.23±0.00b | 0.26±0.05b |
| Cell Motillity | 4.09±0.01a | 3.29±0.41b | 3.53±0.93b | 3.45±0.85ab | 3.22±0.54b |
| Membrane Transport | 14.56±0.02b | 15.75±1.05a | 14.52±1.44b | 15.76±1.63ab | 15.94±1.04a |
| Signal Transduction | 3.45±0.00a | 2.21±0.35c | 2.60±0.74bc | 2.72±0.52b | 2.74±0.44b |
| Replication and Repair | 5.19±0.00c | 6.96±0.45a | 6.55±1.09ab | 6.22±0.74ab | 6.17±0.72b |
| Translation | 3.08±0.00c | 4.36±0.47a | 4.05±0.89ab | 3.74±0.53b | 3.77±0.56b |

Abbreviations: IBA is interbout arousals. Values are means ± SD. Within each row, values not sharing superscripts (a, b, c, and d) differ significantly (*P*-value < 0.05, Kruskal-Wallis’s test)
